# Supplementary material for: Mild Intermittent Cold Stimulation Affects Cardiac Substance Metabolism via the Neuroendocrine Pathway in Broilers
Source: Animals (Basel). 2023 Nov 19;13(22):3577. doi: 10.3390/ani13223577 (PMC10668735; doi:10.3390/ani13223577)
Supplement: Supplementary file 1 [file animals-13-03577-s001.zip › animals-2620566-supplementary.pdf]

Table S1: Primer sequence.

Gene	Series number	Primer sequence (5'-3')
CD36	NM_001030731.1	F: GAAGGTCTGAGCCCAAATGA
		R: AGGTGTCACAAGGAGGTTTAC
EGFR	NM_205497.3	F: TATCCGAGGCCGAACCAAGC
		R: GGTGTCAGCATAGCAGAGGTTCTT
NR4A1	XM_046934299.1	F: CTGCCAGTTCTGCCGCTTCC
		R: AGAGAGGTGATGAGGCTGATGGG
CACNA1C	NM_001396677.1	F: CCAGACATTCACCACGCAGAGAC
		R: GCATTGGAGCCTGAAGATGAGTAGC
AKR1D1	NM_001277393.2	F: TCGCCATATTGACGGTGCCTTTG
		R: GCTCTGGTGGATGACAGGTGTTC
TNFSF10	NM_204379.2	F: ACTCACAGACTTACTTTCGCTTCCG
		R: CTGGACGAGCTGCTTGGGATTC
TPH2	NM_001001301.2	F: CGGAGGGGCTTCTCTCTGGAC
		R: AACTGACTCGCCTTTCCCATTTCC
SESN1	XM_004940327.5	F: TGGTCCGCCTCCTGATGCTG
		R: GATGAATCTGCTTGGTCCGTGTCC
β-actin	NM_205518.1	F: CACCACAGCCGAGAGAGAAAT
		R: TGACCATCAGGGAGTTCATAGC
PI3K	NM_001004410.2	F: CGGATGTTGCCTTACGGTTGT
		R: GTTCTTGTCCTTGAGCCACTGAT
Akt	XM_046941459.1	F: TGATGGCACATTCATTGGCTAC
		R: TGTTTGGTTTAGGTCGTTCTGTCT
mTOR	XM_040689168.2	F: GGACTCTTCCCTGCTGGCTAA
		R: TACGGGTGCCCTGGTTCTG
FOXO1	NM_204328.2	F: AAGGATAAGGGCGACAGCAA
		R: ATTGAGCATCCACCAGGAAC
PEPCK	NM_205471.1	F: GGTTATGATGAGAAGTAGGT
		R: ACAATACACAACAACTGATG
G6P	XM_003642817	F: GCTGCTCACTTTCCTCATC
		R: CCACCTCTGTGCCTTCTC
HK1	XM_040702189.2	F: TGCTGAGCAGAACTGAACTCC
		R: CGGGCAGGTCTTTAGGAGAAG
LDHB	NM_204177.2	F: AAGCAGGTTGTTGAAAGTGC
		R: AAGGCAGGCTCAGGAAGAC
ACC1	NM_205505	F: GCTGGGTTGAGCGACTAATG
		R: GGGAAACTGGCAAAGGACTG
FASN	NM_205155	F: TGAAGGACCTTATCGCATTGC
		R: GCATGGGAAGCATTTTGTTGT
SCD	NM_204890.2	F: CAAGTTCTCCGAGACGCATG
		R: GGGCTTGTAGTATCTCCGCT
*SREBP*1c	NM_204126.3	F: GTCGGCGATCCTGAGGAA

		R: CTCTTCTGCACGGCCATCTT
HSP70	NM_001006685.1	F: CGGGCAAGTTTGACCTAA
		R: TTGGCTCCCACCCTATCTCT
HSP90	NM_001109785.1	F: TCCTGTCCTGGCTTTAGTTT
		R: AGGTGGCATCTCCTCGGT
PFK	NM_204223.2	F: GAGCCACCTGAACATCGTG
		R: CATCACTTCCAGCACAAACG
GLUT1	NM_205209.2	F: GATGGCTTTGTCCTTTGAGATGC
		R: CAAAGATGCTGGTGGAGTAGTAG
